# Food Group and Micronutrient Intake Adequacy among Children, Adults and Elderly Women in Greece

**DOI:** 10.3390/nu7031841

**Published:** 2015-03-11

**Authors:** Yannis Manios, George Moschonis, Evangelia Grammatikaki, Christina Mavrogianni, Ellen GHM van den Heuvel, Rolf Bos, Cecile Singh-Povel

**Affiliations:** 1 Department of Nutrition and Dietetics, Harokopio University of Athens, 70 El Venizelou Avenue, Kallithea 17671, Athens, Greece; E-Mails: gmoschi@hua.gr (G.M.); evagram@hua.gr (E.G.); cmavrog@hua.gr (C.M.); 2 EnviNHealth S.A., Vasilissis Sofias 22, Marousi 151 24, Athens, Greece; 3 FrieslandCampina, Stationsplein 4, Post Box 1551, Amersfoort 3800 BN, the Netherlands; E-Mails: ellen.vandenheuvel@frieslandcampina.com (E.H.); rolf.bos@frieslandcampina.com (R.B.); Cecile.Singh-Povel@frieslandcampina.com (C.S.-P.)

**Keywords:** food groups, EAR, dietary intake, children, adults

## Abstract

The aim of the present study was to record the percentage of children, adults and elderly women in Greece meeting food and micronutrient intake recommendations. Additionally, the present study was aiming to identify the main food contributors of micronutrient intakes and assess the degree up to which meeting food intake recommendations also ensures micronutrient intake adequacy. Dietary intake data from three studies conducted in Greece (on 9–13-year-old children; 40–60-year-old adults; and 50–75-year-old women) were used to estimate mean intakes, the percentages of subjects meeting food and nutrient intake recommendations and the contribution of six core food groups to nutrient intake adequacy. The present study showed that more than 50% of children, adults and elderly women were failing to consume the recommended portions of vegetables, dairy and grains. Furthermore, children and adults consuming the recommended portions of individual core food groups had significantly lower percentages of inadequate micronutrient intakes compared to their counterparts not meeting food intake recommendations (*p* < 0.05). Nevertheless, even among those consuming the recommended portions from a specific core food group, the recommended intake of the corresponding micronutrient (for which this food group is the main contributor) was not always met. Indicatively, 18.2%–44.1% and 4.2%–7.0% of the populations under study were not meeting calcium and vitamin C intake recommendations, although they were consuming the recommended portions of dairy and fruits, respectively. In conclusion, these findings highlight the importance for public health policy makers to take all necessary initiatives to support the population in achieving the recommended intakes from all core food groups, but also emphasize on food variety to ensure adequate intake for all micronutrients.

## 1. Introduction

In Europe, one of the major targets of public health nutrition policy is to control overconsumption of food and calories. However, despite the excess of dietary energy supply, a number of national dietary surveys conducted throughout Europe have consistently reported a considerably increased prevalence of suboptimal micronutrient intakes. Specifically, the synthesis of these dietary intake data by the EURopean micronutrient RECommendations Aligned (EURRECA) Project [1] was indicative of a relatively high (*i.e.*, above 20%) prevalence of inadequate intakes of vitamin C, vitamin D, folate, calcium, selenium and iodine among European adults [2]. Regarding younger age groups, several national or regional dietary surveys conducted with children and adolescents [3,4,5,6] have shown higher intakes of energy and macronutrients, on the one hand, while at the same time, lower intakes of certain micronutrients, such as iron, folate, calcium and vitamin D compared to the recommended levels. Optimal intake of those and other micronutrients is not only important to prevent deficiency-related diseases, but also impacts public health by reducing the risk of non-communicable chronic disease, such as cardiovascular disease [7].

The above “paradox” in terms of having a dietary energy intake surplus, on the one hand, and insufficient dietary intakes of micronutrients on the other, could mainly be due to a shift observed in many developed countries over the last few decades regarding food consumption patterns in their populations. More specifically, the consumption of nutrient-rich core food groups (e.g., whole grains, vegetables and low-fat dairy products) has been partially replaced by the consumption of nutrient-poor, but, at the same time, energy-dense food groups (*i.e.*, added sugars and solid fats) [8]. This shift in food consumption patterns could be considered as one of the main etiological factors of the observed insufficient dietary intakes of micronutrients and seems to be strengthened even more when taking into account the fact that the compliance of the population with food-based dietary recommendations has been repeatedly reported to be particularly low [9,10,11].

Further to the above, any initiative at a public health nutrition policy level aiming to balance dietary intakes of energy and micronutrients should encourage intakes within the recommended thresholds, possibly by putting, among other strategies, special emphasis on adequate consumption of a variety of nutrient-rich core food groups. In this context, the aims of the present study were: (1) to record the percentage of the populations under study meeting the recommended daily portions of six core food groups; (2) to identify which of the core groups are the main contributors of the total dietary intake of 16 essential micronutrients; and (3) whether meeting the recommended daily portions of individual core food groups can also ensure micronutrient intake adequacy.

## 2. Experimental Section

### 2.1. Sampling

Data from three existing studies that took place in Greece, *i.e.*, one large-scale epidemiological study conducted on a representative sample of schoolchildren and two regional studies of a smaller scale conducted on middle-aged adults and postmenopausal women, respectively, were used in the current work. All studies were conducted according to the guidelines laid down in the Declaration of Helsinki, and all procedures involving human subjects were approved by the Ethics Committee of Harokopio University (Ethics approval code for the Healthy Growth Study: 16/19-12-2006; Ethics approval code for both the CardioHealth Study and the Postmenopausal Health Study II: 18/01-11-2007). Written informed consent was obtained from all subjects and from parents in the case of children.

#### 2.1.1. Healthy Growth Study

The ‘Healthy Growth Study’ (HGS) was a cross-sectional epidemiological study initiated in May 2007. In addition to the approval granted by the Harokopio University Ethics Committee, permission to conduct the study was also granted by the Greek Ministry of National Education. The study population comprised schoolchildren (9–13 years old) attending the 5th and 6th grades of primary schools located in municipalities within the wider regions of four Greek counties, and it was representative at a county level. The vast majority of these children were aged 10–12 years (88.9%), while the remaining study sample (11.1%) included some younger (*i.e.*, 9–10 years) and older (*i.e.*, 12–13 years) children. The sampling procedure is described in detail elsewhere [12]. In brief, the sampling of schools was random, multistage and stratified by parental educational level and by the total population of students attending schools within these municipalities. An extended letter explaining the aims of the present study and a consent form for conducting full measurements were provided to all parents or guardians having a child in these schools. Signed parental consent forms were collected for 2660 out of 4145 children (response rate: 64%). The current analysis included 1100 children with full dietary intake data on energy, macro- and micro-nutrients, as well as on food group consumption.

#### 2.1.2. CardioHealth Study

In October 2007, an open invitation for participation in the study was advertised, and a total of 340 men and women from the wider district of Athens volunteered to participate. Based on the eligibility criteria used for the participation of volunteers in the current study, described elsewhere [13], 154 middle-aged men and women (40–60 years old) were considered eligible, and full dietary intake data were collected. Written informed consent was obtained from all study participants.

#### 2.1.3. Postmenopausal Health Study II

In March 2008, volunteers were invited to participate via informational brochures and posters distributed in public buildings and community centers in municipalities from the wider district of Athens. The study protocol registration number was NTR1396. Through the initial screening of the study, a sample of 720 postmenopausal women volunteered to participate. Based on the eligibility criteria used for participation of volunteers in the current study, described elsewhere [14], 214 eligible women (50–75 years old) were invited to participate in the study and full dietary intake data were collected. Written informed consent was obtained from all study participants.

### 2.2. Dietary Intake

Dietary intake data were obtained by trained dietitians and nutritionists via morning interviews with children at the school site (in the case of HGS) and with adult study participants during scheduled meetings at Harokopio University in the case of the CardioHealth study and the Postmenopausal Health Study II. Dietary intake data were obtained for two consecutive weekdays and one weekend day in all three studies using the 24-h recall technique. To ensure quality, food models and sample household measurements (such as cups and spoons) were used to specify serving sizes; a trained dietitian checked the food records for any misrecorded or missing information, while a random 10% of all records was re-entered in the food database by a different person than the one entering the original data. The data were then compared with the original to ensure inter-researcher reliability. Food intake data were analyzed with Nutritionist V diet analysis software (version 2.1, 1999, First Databank, San Bruno, CA, USA), which was extensively amended to include traditional Greek foods and recipes, as described in the Food Composition Tables and Composition of Greek Cooked Food and Dishes [15,16]. Furthermore, the database was also updated with commercially available food products widely consumed by children and adults in Greece and their nutrient content, as derived by available information from food labels, as well as chemical analyses of these food items.

The distribution of usual intakes was estimated by using the National Research Council method (NRC method), which attempts to remove the effects of day-to-day (within person) and person-by-person (between persons) variability in dietary intakes [17] and represents a standard procedure mitigating this main methodological limitation stemming from using 24-h recalls to assess dietary intakes. Furthermore, under reporters, *i.e.*, study participants under reporting their dietary energy and, consequently, nutrients and food intake, were also identified. To identify under reporters, the age and sex-specific cut-off points proposed by Goldberg *et al*. [18] for the ratio of reported energy intake and predicted basal metabolic rate (estimated by Schofield equations [19]) were used. This is a population-based method that takes into account the size of the sample population and the number of days of dietary intake records for the detection of under-reporters. The identified under-reporters from all three cohorts were not included in the analyses conducted in the current study.

The estimated average requirement (EAR) cut-off point method proposed by the Institute of Medicine (IOM) was used to assess the adequacy of the nutrient intakes of children, adults and elderly women [20,21]. The rationale of using IOM’s dietary intake reference values was based on the fact that these are regularly updated and frequently used compared to reference values provided by other scientific bodies or organizations. For micronutrients with an EAR, the EAR threshold was used to estimate the proportion of each one of the three populations with usual intakes below the EAR [20,22]. For only one micronutrient (*i.e.*, potassium), for which there are no established EAR values, the proportion below the adequate intake (AI) threshold was used alternatively. In order to define the inadequacy of nutrient intake by the populations under study, a threshold of 20% was set with respect to the percentage of subjects having intakes below the EAR values. The rationale for using this specific threshold was based on the available literature from the EURRECA project reporting inadequate nutrient intakes for adults in Europe [2].

### 2.3. Food Grouping

A food-grouping scheme was designed for all foods or entries (core and recipes) appearing in Nutritionist V. The total number of individual foods consumed were grouped into the following six core food groups based on the food grouping scheme provided by the USDA ChooseMyPlate.gov [23]: (i) dairy; (ii) protein foods (*i.e.*, meat, poultry, seafood, eggs, nuts and seeds and legumes); (iii) fruits (whole fruit and fruit juices); (iv) vegetables (starchy vegetables included); (v) grains (*i.e.*, cereals, cereal products and other starchy foods); and (vi) oils. Composite/complex food items and recipes were analyzed for their ingredients, which were further allocated to their relevant core food group. Table 1 summarizes the portion size of individual food items within each one of the six core food groups identified in the present study based on the USDA MyPlate guidelines [23]. In the case of soft and hard cheeses, national adaptations were made with regards to portion sizes in order to increase the consistency with food intake recommendations and practices in Greece. More specifically, a portion size of 30 g was used for hard cheeses, including traditional Greek ones (*i.e.*, feta, graviera, kefalotyri, kasseri, *etc*.) instead of 42.5 g (or 1.5 ounces) as proposed by the USDA MyPlate guidelines [23]. Regarding soft cheeses, including traditional Greek ones (*i.e.*, anthotyro, mizithra, *etc*.), a portion size of 45 g proposed by the USDA 2005 guidelines [24] was used instead of 56.7 g (or 2 ounces) proposed by the USDA MyPlate guidelines [23].

Following the calculation of the number of portions consumed from each one of the six core food groups, study participants in all three cohorts were dichotomized based on whether they were meeting or not the relevant USDA MyPlate dietary intake recommendations [23].

### 2.4. Contribution of Food Groups in Micronutrient Total Dietary Intake

The percent contribution of the six core food groups to the total dietary intake of each one of the 16 micronutrients under study was also calculated. Those food groups contributing to more than 30% of a micronutrient’s total dietary intake were considered to be its “main food sources”.

**Table 1:** Recommended portions and portion sizes of individual food items within the six core food groups based on the USDA MyPlate guidelines [23].

Food Group	Recommended Portions		Portion Size
Dairy	All age groups under study	3 portions (cups)	1 cup of milk, 30 g ^†^ of hard cheese (*i.e.*, feta, edam/gouda, cheddar, kasseri, parmesan, graviera and mozzarella), 45 g ^†^ of soft cheeses (*i.e.*, anthotyro; mizithra) and cream cheese and 1 regular container of yogurt
Protein Foods	Children and all women under study	5 portions (equivalents)	1 ounce of meat, poultry or seafood, 1 egg, 0.5 ounces of nuts or seeds, 0.25 cup of cooked legumes *
	Men 31–50 years	6 portions (equivalents)	
	Men 51+ years	5.5 portions (equivalents)	
Fruits	Children and all women under study	1.5 portions (cups)	1 cup of fresh fruit, 0.5 cups of dried fruit, 1 cup of fresh fruit juice.
	All adult men	2 portions (cups)	
Vegetables	Girls and women 51+ years	2 portions (cups)	1 cup of raw or cooked vegetables or vegetable juice, 2 cups of raw leafy greens
	Boys, women 31–50 years and men 51+ years	2.5 portions(cups)	
	Men 31–50 years	3 portions (cups)	
Oils	Children and adult women	5 portions (teaspoons)	1 teaspoon of vegetable oils (such as olive, canola, corn, cottonseed, peanut, safflower, soybean and sunflower oil) or soft margarine
	Adult men	6 portions (teaspoons)	
Grains	Girls and women 51+ years	5 portions (equivalents)	1 ounce equivalent of grains, such as a regular slice of bread, 0.5 cups cooked pasta or rice, 1 cup flakes or rounds
	Boys, women 31–50 years and men 51+ years	6 portions (equivalents)	
	Men 51+ years	7 portions (equivalents)	

^†^ This portion size is not the one proposed by the USDA MyPlate guidelines [23], but it has been adapted to the food intake recommendations and practices followed in Greece; * legumes are considered part of the protein foods; however, after the suggested intake level in the Protein Foods Group is reached, any additional legumes eaten are counted as part of the vegetable group.

### 2.5. Statistical Analysis

Continuous variables are expressed as means (standard deviations (SD)) and as medians (interquartile ranges (IQR)), whereas categorical variables are expressed as percentages (%). The Kolmogorov-Smirnov test was used to determine the normality of the distribution of the examined variables. The chi-square test was used to explore the association between categorical variables, using the two-sample *z*-test for proportions for *post hoc* comparisons. The Student’s *t*-test and, whenever appropriate, the non-parametric Mann-Whitney test were used for the comparison of mean values between groups. All *p*-values reported are two-tailed. Statistical analysis was done with SPSS Version 21.0. The level of statistical significance was set at *p* < 0.05.

## 3. Results

Inadequate intakes were observed for several core food groups (Table 2). Specifically, more than 50% of children, middle-aged adults and postmenopausal were not meeting the consumption of the recommended portions for dairy, vegetables and grains. Similar patterns of inadequate consumption from the six core food groups were observed for 11–13-year-old girls, middle-aged adults and postmenopausal women regarding the consumption of protein foods; and 9–10-year-old girls, 11–13-year-old children and middle-aged adults regarding the consumption of oils. As far as gender differences were concerned, 9–10-year-old boys were consistently reported to have a higher average consumption of portions from almost all food groups (with the only exception being the consumption of fruits) compared to girls. On the contrary, the relevant percentages of 9–10-year-old children not meeting the consumption of the recommended portions from dairy and oils were higher in girls compared to boys.

Regarding micronutrients, inadequate intakes (*i.e.*, more than 20% of study participants below the age- and sex-specific EAR thresholds) were observed in all age groups for calcium, magnesium, potassium, folate, vitamin E and vitamin D. Specifically, concerning vitamin D, 100% of study participants in all age groups were found to have dietary intakes below the EAR threshold. Furthermore, dietary intakes were found to be inadequate for vitamin A in both children and middle-aged adults and for zinc and vitamins B1, B6, B12 and C in both middle-aged adults and postmenopausal women. In addition, copper and iron intakes were found to be inadequate only in middle-aged women, while selenium intake was found to be inadequate only in the case of children. As far as gender differences were concerned, the average intake for the majority of nutrients was found to be almost consistently higher for males compared to females, while the relevant percentages with inadequate dietary intakes were found to be higher for females compared to males. More detailed information on the age- and sex-specific differences observed in the present study have been described elsewhere [25].

Table 3 summarizes the mean percent contribution of each food group to the intake of each one of the 16 micronutrients of interest. The “main food sources” of micronutrients have been highlighted in bold (*i.e.*, those food groups contributing to at least 30% of the total micronutrient intake). More specifically, dairy was the “main food source” of calcium, vitamin B2, vitamin B12 and vitamin D in all age groups under study. Protein foods were the “main food sources” of vitamin B12 in all age groups, of selenium and zinc in both children and middle-aged adults and of iron (*i.e.*, both heme and non-heme from animal and plant foods, respectively), vitamin B1 and vitamin B6 in children. Fruits were the “main food source” of vitamin C in all age groups. Vegetables were found to be the “main food source” of vitamin A and vitamin C in middle-aged adults and postmenopausal women and of folate in postmenopausal women. Oils were the “main food sources” of vitamin E in all age groups. Lastly, grains were found to be the “main food sources” of iron and vitamin B1 in all age groups, of selenium in middle-aged adults and postmenopausal women and of folate in children and middle-aged adults.

If a food group was the “main food source” of a specific micronutrient, the percentage of inadequate intake for this specific micronutrient for children and adults meeting or not the recommended consumption of its “main food source” is presented in Table 4. In all food groups, the percentages of study participants with dietary intakes of calcium, vitamin B2 and vitamin B12 below EAR were found to be significantly lower for those meeting compared to those not meeting the recommended portions of dairy consumption. Furthermore, in those study participants meeting the recommended portions of protein foods, the percentages below EAR were found to be lower for the dietary intakes of selenium and zinc in both children and middle-aged adults; vitamin B1 and B6 only in children; and vitamin B12 in both children and postmenopausal women; compared to their counterparts not meeting the recommended portions of protein foods. Regarding fruit intake, the percentages of study participants in all age groups with dietary intakes of vitamin C below EAR were found to be lower for those consuming compared to those not consuming the recommended portions. In addition, in study participants consuming the recommended portions for vegetables, the percentages below EAR were found to be lower for the dietary intakes of vitamin A in postmenopausal women and vitamin C in both middle-aged adults and postmenopausal women compared to those study participants not consuming the recommended portions. Regarding oils, the percentages of children with dietary intakes of vitamin E below EAR were found to be lower for those consuming compared to those not consuming the recommended portions. Lastly, in those study participants consuming the recommended portions of grains, the percentages below EAR were found to be lower for the dietary intakes of iron in middle-aged adults; vitamin B1 in both middle-aged adults and postmenopausal women; and folate in children and middle-aged adults; compared to their counterparts not consuming the recommended portions of grains.

**Table 2:** Mean (and median) consumption of core food groups and percentages of children, middle-aged adults and postmenopausal women in Greece with consumption below the recommended ^†^ portions for each one of these food groups ^‡^.

Food Groups	9–10 Years		11–13 Years Old		40–60 Years Old		50–75 Years Old
	Males	Females	Males	Females	Males	Females	Females
	(*n* = 264)	(*n* = 260)	(*n* = 277)	(*n* = 299)	(*n* = 79)	(*n* = 75)	(*n* = 214)
	Mean (SD)	Mean (SD)	Mean (SD)	Mean (SD)	Mean (SD)	Mean (SD)	Mean (SD)
	Median (IQR)	Median (IQR)	Median (IQR)	Median (IQR)	Median (IQR)	Median (IQR)	Median (IQR)
	% < Recommended	% < Recommended	% < Recommended	% < Recommended	% < Recommended	% < Recommended	% < Recommended
	Portions	Portions	Portions	Portions	Portions	Portions	Portions
Dairy	2.81 (1.80)	2.25 (1.56) *	2.59 (1.95)	2.45 (1.66)	2.32 (1.58)	1.94 (1.38)	2.02 (1.28)
	2.77 (1.73–3.79)	2.24 (0.92–3.37) *	2.50 (0.93–3.77)	2.37 (0.94–3.57)	2.07 (1.32–3.06)	1.82 (1.05–2.50)	1.86 (1.20–2.67)
	56.80%	69.60%	61.90%	61.70%	75.00%	85.40%	84.10%
Protein Foods	7.14 (5.63)	5.86 (4.62) *	6.50 (5.22)	5.39 (4.67) *	4.74 (2.91)	3.80 (2.26)	2.98 (2.08)
	5.99 (3.02–9.99)	5.00 (3.31–8.40) *	5.05 (2.83–9.05)	3.85 (1.99–7.72) *	4.02 (2.46–6.48)	3.47 (1.93–5.39)	2.61 (1.47–4.09)
	41.70%	49.80%	49.10%	56.70%	71.70%	70.80%	83.20%
Fruits	1.57 (1.43)	1.61 (1.34)	1.48 (1.36)	1.63 (1.62)	1.46 (1.67)	1.24 (1.07)	1.88 (1.93)
	1.23 (0.48–2.31)	1.40 (0.69–2.15)	1.22 (0.45–2.19)	1.30 (0.64–2.29)	0.85 (0.08–1.90)	0.98 (0.37–2.09)	1.63 (0.88–2.45)
	56.10%	53.60%	57.50%	58.00%	78.30%	66.70%	46.30%
Vegetables	1.21 (0.98)	1.00 (0.97) *	1.18 (1.11)	1.03 (0.95)	1.90 (1.14)	1.83 (1.00)	1.99 (1.47)
	0.98 (0.51–1.72)	0.76 (0.35–1.35) *	0.89 (0.44–1.72)	0.80 (0.34–1.40)	1.84 (1.13–2.51)	1.66 (1.09–2.33)	1.82 (1.14–2.49)
	91.30%	89.00%	93.00%	86.0% ^§^	83.30%	75.00%	58.90%
Oils	6.13 (4.06)	5.20 (3.80) *	5.28 (3.74)	5.05 (3.96)	6.16 (3.88)	5.22 (2.76)	6.41 (3.27)
	5.57 (3.06–8.72)	4.45 (2.56–7.13) *	4.96 (2.22–7.41)	4.14 (2.25–7.35)	5.80 (3.85–8.07)	4.58 (3.20–7.12)	5.77 (4.04–8.28)
	45.50%	57.0% ^§^	50.50%	58.30%	53.30%	56.30%	36.40%
Grains	5.03 (2.53)	4.27 (2.27) *	4.93 (3.16)	4.62 (2.76)	5.82 (2.95)	3.68 (1.70) *	3.66 (1.61)
	4.76 (3.34–6.11)	4.10 (2.67–5.41) *	4.49 (2.81–6.72)	4.04 (2.73–5.95)	5.74 (3.39–7.76)	3.46 (2.56–4.64) *	3.58 (2.57–4.52)
	73.50%	67.70%	68.10%	63.00%	66.70%	85.4% ^§^	82.20%

^‡^ Foods were grouped into the following six food groups based on the food grouping as indicated by MyPlate (UDSA) [23]: (i) dairy (*i.e.*, milk, cheese and yogurt); (ii) protein foods (*i.e.*, meat, fish, poultry, eggs and legumes, nuts and seeds); (iii) fruits (whole fruit and fruit juices); (iv) vegetables (starchy vegetables included); (v) grains (*i.e.*, cereals, cereal products and other starchy foods); and (vi) oils. ^†^ Recommended daily consumption: (i) dairy, three portions (cups) in all age groups under study; (ii) protein foods, five portions (equivalents) for children and adult women/six portions (equivalents) for men 31–50 years and 5.5 portions (equivalents) for men 51+ years; (iii) fruits, 1.5 portions (cups) for children and adult women/two portions for adult men; (iv) vegetables, two portions (cups) for girls and women 51+ years/2.5 portions (cups) for boys, women 31–50 years and men 51+ years; three portions (cups) for men 31–50 years; (v) grains, five portions (equivalents) for girls and women 51+ years/six portions (equivalents) for boys, women 31–50 years and men 51+ years/seven portions (equivalents) for men 30–50 years; (vi) oils, five portions (teaspoons) for children and adult women/six portions (teaspoons) for adult men (based on the USDA MyPlate [23]). * *p*-value < 0.05 for the comparisons of mean values between males and females using Student’s *t*-test and Mann–Whitney test whenever appropriate. ^§^ *p*-value < 0.05 for the comparisons of percentages between males and females using the chi-square test.

**Table 3:** Percent contribution of core food groups in the total dietary intake of micronutrients by children, middle-aged adults and postmenopausal women in Greece.

Core Food Groups	Calcium (mg/day)	Copper (μg/day)	Iron (mg/day)	Magnesium (mg/day)	Potassium (g/day)	Selenium (μg/day)	Zinc (mg/day)	Vitamin A (μg/day)	Vitamin B1 (mg/day)	Vitamin B2 (mg/day)	Vitamin B6 (mg/day)	Vitamin B12 (μg/day)	Folate (μg/day)	Vitamin C (mg/day)	Vitamin D (μg/day)	Vitamin E (mg/day)
	%	%	%	%	%	%	%	%	%	%	%	%	%	%	%	%
**Children and Adolescents 9–13 Years Old (Healthy Growth Study) (*n* = 1100)**																
Dairy	**63.2**	3.0	9.0	22.8	26.0	28.6	28.1	26.1	9.6	**42.9**	17.3	**47.2**	6.4	5.3	**31.0**	6.5
Protein Foods	5.3	27.0	**30.9**	21.9	24.9	**34.3**	**36.5**	20.5	**32.6**	29.5	**35.0**	**40.1**	20.9	3.6	20.2	15.1
Fruits	3.1	16.0	5.9	11.5	17.3	6.0	3.2	6.7	11.5	5.1	13.1	1.0	17.5	**48.2**	0.0	5.6
Vegetables	3.2	17.6	9.3	11.4	16.0	5.5	4.1	25.2	9.7	4.6	12.5	0.0	20.5	27.9	0.0	7.2
Oils	0.1	0.0	0.8	0.0	0.1	0.0	0.1	4.3	0.0	0.1	0.0	0.1	0.0	0.0	16.0	**43.2**
Grains	11.3	28.2	**37.2**	23.3	8.7	25.2	20.6	7.6	31.9	15.2	21.0	10.8	**34.1**	10.2	16.4	11.3
**Adults 40–60 Years Old (CardioHealth Study) (*n* = 154)**																
Dairy	**57.2**	2.9	7.7	13.8	14.3	12.6	26.3	23.5	8.9	**37.2**	14.5	**47.9**	8.0	3.1	**37.1**	3.6
Protein Foods	7.7	20.2	24.8	19.2	19.5	**36.7**	**38.2**	23.8	24.6	24.4	29.1	**45.5**	20.8	1.6	26.7	15.0
Fruits	4.3	9.4	3.4	6.9	12.8	1.5	2.2	6.5	9.7	4.6	10.6	0.0	11.4	**38.6**	0.0	6.3
Vegetables	11.4	23.7	17.0	17.2	26.3	1.8	8.0	**40.5**	16.7	11.4	24.3	0.2	25.9	**50.1**	0.0	15.0
Oils	0.1	0.0	1.0	0.0	0.0	0.0	0.2	3.5	0.0	0.1	0.0	0.1	0.0	0.0	16.4	**47.3**
Grains	14.8	22.5	**37.7**	21.9	9.4	**44.8**	19.5	2.0	**40.1**	20.3	14.0	3.4	**32.0**	1.3	10.4	8.9
**Postmenopausal Women 50–65 Years Old (Postmenopausal Health Study II) (*n* = 214)**																
Dairy	**58.7**	2.3	10.8	16.4	17.6	15.2	27.4	16.0	9.9	**41.0**	17.0	**33.4**	8.1	2.9	**49.5**	2.6
Protein Foods	5.7	17.9	19.9	14.6	13.6	25.5	29.0	12.9	17.4	18.5	21.4	**58.9**	14.4	1.1	9.3	7.8
Fruits	4.6	18.8	7.8	11.2	20.0	1.1	3.8	8.5	16.6	8.9	17.8	0.1	12.2	**39.7**	0.0	13.8
Vegetables	9.5	23.8	17.9	19.6	26.9	2.1	8.3	**54.1**	19.4	11.6	23.5	0.0	**36.8**	**45.4**	0.0	13.1
Oils	0.1	0.0	1.2	0.0	0.1	0.0	0.3	3.9	0.0	0.1	0.0	0.2	0.0	0.0	23.9	**50.1**
Grains	11.2	21.6	**33.0**	20.5	7.6	**52.3**	19.9	2.3	**32.5**	14.0	13.2	5.7	27.8	1.4	7.9	7.6

Figures in bold correspond to the core food groups that contribute >30% to the intake of the specific micronutrient, indicating the “major food sources” of this micronutrient.

**Table 4:** Percentages of children, middle-aged adults and postmenopausal women with intakes below EAR for certain micronutrients in those subjects meeting and those not meeting the recommended ^†^ consumption of the food group that contributes the most to the intake of the specific micronutrient.

*Food Group*	Children and Adolescents 9–13 Years Old			Adults 40–60 Years Old			Postmenopausal Women 50–65 Years Old (Postmenopausal Health Study II)		
	(Healthy Growth Study)			(CardioHealth Study)					
	Not Meeting	Meeting		Not Meeting	Meeting		Not Meeting	Meeting	
	Recommendations	Recommendations		Recommendations	Recommendations		Recommendations	Recommendations	
	% < EAR	% < EAR	*p*-value	% < EAR	% < EAR	*p*-value	% < EAR	% < EAR	*p*-value
*Dairy*									
Calcium	**78.6**	**21.8**	**<0.001**	**59.3**	**18.2**	**0.001**	**96.7**	**44.1**	**<0.001**
Vitamin B2	**2.8**	**0.0**	**0.001**	**31.4**	**9.1**	**0.035**	**16.1**	**0.0**	**0.012**
Vitamin B12	**3.6**	**0.0**	**<0.001**	**38.4**	**13.6**	**0.028**	**-**	**-**	**-**
Vitamin D	100.0	100.0	-	100.0	100.0	-	100.0	100.0	-
*Protein Foods*									
Iron	2	1.3	0.319	-	-		-	-	
Selenium	**61.3**	**51.9**	**0.002**	**13.0**	**0.0**	**0.035**	**-**	**-**	
Zinc	**16.0**	**4.0**	**<0.001**	**58.4**	**35.5**	**0.031**	**-**	**-**	
Vitamin B1	**11**	**3.4**	**<0.001**	**-**	**-**		**-**	**-**	
Vitamin B6	**4.1**	**0.4**	**<0.001**	**-**	**-**		**-**	**-**	
Vitamin B12	**3.7**	**0.9**	**0.002**	35.1	29.0	0.547	**35.4**	**11.1**	**0.004**
*Fruits*									
Vitamin C	**28.3**	**4.2**	**<0.001**	**51.9**	**0.0**	**<0.001**	**38.4**	**7.0**	**<0.001**
*Vegetables*									
Vitamin A	-	-		45.3	36.4	0.448	**15.1**	**2.3**	**0.002**
Folate	-	-		-	-		97.6	96.6	0.654
Vitamin C	**-**	**-**		**46.5**	**4.5**	**<0.001**	**33.3**	**4.5**	**<0.001**
*Oils*									
Vitamin E	**87.1**	**60.1**	**<0.001**	88.1	79.6	0.225	96.2	94.1	0.516
*Grains*									
Iron	2.1	0.6	0.056	**21.0**	**3.7**	**0.037**	2.3	0.0	0.348
Selenium	-	-		11.1	3.7	0.250	6.3	0.0	0.111
Vitamin B1	7.6	6.3	0.415	**53.1**	**29.6**	**0.034**	**61.9**	**26.3**	**<0.001**
Folate	**65.3**	**52.7**	**<0.001**	**86.4**	**48.1**	**<0.001**	-	-	

Figures in bold highlight statistically significant differences in the percentages of study participants with intakes of specific micronutrients below EAR, between those meeting and those not meeting the recommended consumption of the food group that contributes >30% to the intake of the specific micronutrient. ^†^ Recommended daily consumption: (i) dairy, three portions (cups) in all age groups under study; (ii) protein foods, five portions (equivalents) for children and adult women/six portions (equivalents) for men 31–50 years and 5.5 portions (equivalents) for men 51+ years; (iii) fruits, 1.5 portions (cups) for children and adult women/two portions for adult men; (iv) vegetables, two portions (cups) for girls and women 51+ years/2.5 portions(cups) for boys, women 31–50 years and men 51+ years; three portions (cups) for men 31–50 years; (v) grains, five portions (equivalents) for girls and women 51+ years/six portions (equivalents) for boys, women 31–50 years and men 51+ years/seven portions (equivalents) for men 30–51 years; (vi) oils, five portions (teaspoons) for children and adult women/six portions (teaspoons) for adult men (based on the USDA MyPlate [23]).

## 4. Discussion

The current study analyzed food and nutrient intake data in a large representative sample of schoolchildren and two smaller regional samples of adults in Greece. One of the main findings was that the consumption of the examined core food groups was below recommendations for the vast majority of children and adults. That could be one of the main reasons explaining the relatively high percentages of inadequate micronutrient intakes observed in the present study and discussed in detail elsewhere [25]. Regarding micronutrient intakes, the findings of the current study showed some clear sex- and age-specific differences, with intakes for the majority of the examined micronutrients being higher in females compared to males and in postmenopausal women compared to younger age groups. Regarding consumption of food items from the six core food groups, the lowest compliance with the recommendations was mainly observed for vegetables, followed by dairy and grains (Table 2). More specifically, the current study showed that the percentage of children under study not meeting the recommended portions for vegetables was considerably high (*i.e.*, 93% in 11–13-year-old boys). The neutral or sour taste of vegetables possibly explains this very low consumption by making this food group less appealing to children [26,27]. On the other hand, inadequate consumption of dairy and grains was found to increase with age, being higher in middle-aged and postmenopausal women compared to children. The high prevalence of lactose intolerance, reported among Greek adults [28], possibly discourages them from including dairy, especially milk, in their daily diet plan.

The link between the high prevalence of inadequate nutrient intakes observed in the current study with the very low consumption of nutrient-rich core food groups can be further supported by the data presented in Table 3 regarding the contribution of food groups to the intake of the examined micronutrients. The data presented in Table 3 is very much comparable to the relevant data presented by the Dutch National Food Consumption Survey (2007–2010), such as the major contribution of dairy to the dietary intakes of calcium, vitamin B2 and vitamin B12 [29]. Nonetheless, there were also several notable differences probably attributed to both methodological issues, as well as to the different dietary patterns between Greece and the Netherlands. The data presented in the current study indicates that consumption of a variety of food items from core food groups is probably the optimal approach in tackling dietary intake inadequacies. In this context the USDA My Plate recommendations have been developed focusing mostly on nutrient-dense foods and limiting solid fats and added sugars, as well as alcohol (in adults), aiming to cover essential nutrient needs without exceeding total energy needs [30]. The above indicates that if consumption of the recommended portions of core food groups is not met, then the dietary intake of micronutrients remains very low, and consequently, the prevalence of inadequacies and the risk of micronutrient insufficiencies increase.

Taking the above into consideration, the data presented in Table 4 is an attempt to provide the prevalence of inadequacy in micronutrient intakes when the consumption of the recommended portions from core food groups is either met or not. This data support the observation that when the recommended portions of core food groups (major intake contributors of specific vitamins and minerals) are met, then the prevalence of the population with inadequate intakes is considerably low. Nevertheless, according to the same data, meeting food intake recommendations just for one food group does not completely eliminate inadequate micronutrient intakes. The implication of this observation is that micronutrient intake adequacy is ensured when consuming the recommended portions from all core food groups and not focusing only on the consumption of specific food groups. For example, although dairy is the major food source for calcium, vitamin B2 and vitamin B12 in both children and adults, meeting the recommended portions of milk and milk products [31] does not guarantee adequacy in the dietary intake of these micronutrients. However, their combined consumption with other foods, such as whole and fortified grains (e.g., through breakfast cereals) could considerably increase the likelihood of meeting nutrient intake recommendations in all of these essential micronutrients.

In the same context as above, although the present study has highlighted dairy as the main food source of dietary vitamin D, still, the prevalence of inadequate vitamin D intake was 100% regardless of sex, age or meeting the recommended portions for the consumption of dairy. Despite the fact that Greece is a Mediterranean country with quite a lot of sunlight (*i.e.*, the main source of endogenous vitamin D synthesis) throughout the year, the reports of a high prevalence of vitamin D insufficiency [32] are indicative of the important role of diet in tackling this health issue. Considering that the natural food sources of dietary vitamin D are apparently not adequate to ensure adequate intakes of this important vitamin, food fortification combined with supplementation could be one approach for tackling vitamin D insufficiency. This argument is probably strengthened when considering the much lower prevalence rates of vitamin D insufficiency in both children and adults living in Nordic countries compared to southern European countries [33,34]. Mandatory vitamin D food fortification, as well as the much higher use of supplements by populations living in northern compared to southern European countries (*i.e.*, 40% *vs*. 5%) [35] could be used not only for interpreting the results of the current and previous studies [36] on vitamin D, but also for facilitating future research and possibly discussions on vitamin D food fortification policy in southern Europe.

The findings of the current study should be interpreted in light of its strengths and limitations. Regarding strengths, adjustments for the effects of day-to-day (within-subject) and subject-by-subject (between-subject) variability to estimate usual intakes as well as the exclusion of subjects that were under-reporting their food consumption could be considered as the strongest component of the methodological approach used in the present study. Furthermore, the representativeness of the sample in the HGS and the use of the same methods and procedures to record dietary intake in all studies are additional strong points of the present work. Regarding limitations, the findings derived from the two studies conducted on adults (*i.e.*, in the CardioHealth and the Postmenopausal Health Study II) cannot be generalized to the wider adult population in Greece, since both study samples were representative only for the metropolitan area of Athens. Furthermore, although food frequency data could offer important covariate information in supplementing multiple recalls for estimating usual dietary intakes [37], relevant food frequency data were not available for the three populations under study and, as such, could not be used in the estimation of usual intakes in the current work. Lastly, the use of non-national food and nutrient intake recommendations (*i.e.*, USDA and IOM’s, respectively) for assessing the adequacy in the dietary intakes of food groups and nutrients, respectively, could also have affected, up to a certain extent, the findings regarding the prevalence of inadequate intakes. In this context, when interpreting the current findings, it should be kept in mind that these are dietary intake data and, as such, can provide information only on inadequate nutrient intakes, but not on nutrient deficiencies, since no such biological markers were assessed in the current study.

## 5. Conclusions

The present study reported a considerably high prevalence of inadequate dietary intakes for the vast majority of the core food groups and essential micronutrients examined. Furthermore, inadequate dietary intake of vitamin D by 100% of children and adults in the three populations under study regardless of sex, age or meeting food intake recommendations requires special attention by public health authorities. In addition, the current study showed that each one of the food groups of USDA My Plate was the main source for a different set of micronutrients. Nevertheless, meeting food intake recommendations just for one food group does not safeguard adequate intakes, even for those micronutrients for which this food group is considered a main contributor. This indicates the need to meet recommendations for all core food groups in order to ensure adequate intakes of all micronutrients. Overall, the findings of the present study should be taken into consideration by public health and nutrition policy makers in taking all necessary initiatives to support the population in achieving the recommended intakes from all core food groups, but also emphasize food variety and consider, wherever needed, food fortification strategies [38] prioritizing micronutrients with very low dietary intakes.
